# Association Between Obstetric Conjugate Diameter Measured by Transabdominal Ultrasonography During Pregnancy and the Type of Delivery

**DOI:** 10.5812/iranjradiol.13191

**Published:** 2013-08-30

**Authors:** Mohammad Hossein Daghighi, Masoud Poureisa, Mahnaz Ranjkesh

**Affiliations:** 1 Department of Radiology, Radiotherapy and Nuclear Medicine, Tabriz University of Medical Sciences, Tabriz, Iran; 2 Neurosciences Research Center (NSRC), Tabriz University of Medical Sciences, Tabriz, Iran

**Keywords:** Ultrasonography, Pelvimetry, Cesarean Section

## Abstract

**Background:**

Normal morphological features of the maternal pelvis are an important prerequisite to vaginal delivery.

**Objectives:**

We aimed to evaluate the association between obstetric conjugate diameter (OCD) measured by ultrasonography and the type of delivery, vaginally (V) or by cesarean (C) section.

**Patients and Methods:**

Pelvimetry was performed in 200 primigravid women for fetal cephalic presentation. The OCD was measured twice by transabdominal ultrasonography during 25-30 weeks and 30-35 weeks of pregnancy.

**Results:**

The mean OCD of both sonographies in groups V and C was 125.51± 8.35 mm (105-144.5) and 112.99 ± 8.53 mm (96-134.5), respectively, which was significantly lower in group C (P<0.001). The values of OCD between the first and second measurements were not different significantly (P=0.065). C-section was indicated in 65 (32.5%) mothers. The optimal cut-off point for the OCD in the prediction of vaginal delivery was ≥ 119.75 mm, with a sensitivity and specificity of 80% and 78.5%, respectively.

**Conclusion:**

The US measurement of OCD might be an accurate method that almost always remains constant during late pregnancy; it is easy to measure and might be confidentially employed for predicting C-section, but needs more precise studies to be used widely.

## 1. Background

Cesarean is a word usually used in midwifery to describe live embryo parturition through laparotomy and hysterotomy. Cesarean progressively increased in USA from 1965 to 1988 (1). C-section rate has been steadily rising from 35% in 2000 to 40% in 2005 in Iran (2). Meanwhile, the size and shape of the bony pelvis are important factors determining the progress of labor and delivery. Most morphological disorders of the pelvis result from a small anterior-posterior diameter or obstetric conjugate diameter (OCD) (3). Different methods are used to measure this diameter including clinical examination, X-ray pelvimetry, CT scan and MRI (4). Ultrasound (US) is another technique to measure OCD. It is also accessible in many centers and considered as an exact way to determine OCD (4, 5).

## 2. Objectives

Our study aims to measure OCD by transabdominal sonography during late pregnancy and assessing its association with the type of parturition in Iranian mothers.

## 3. Patients and Methods

**Figure 1.**
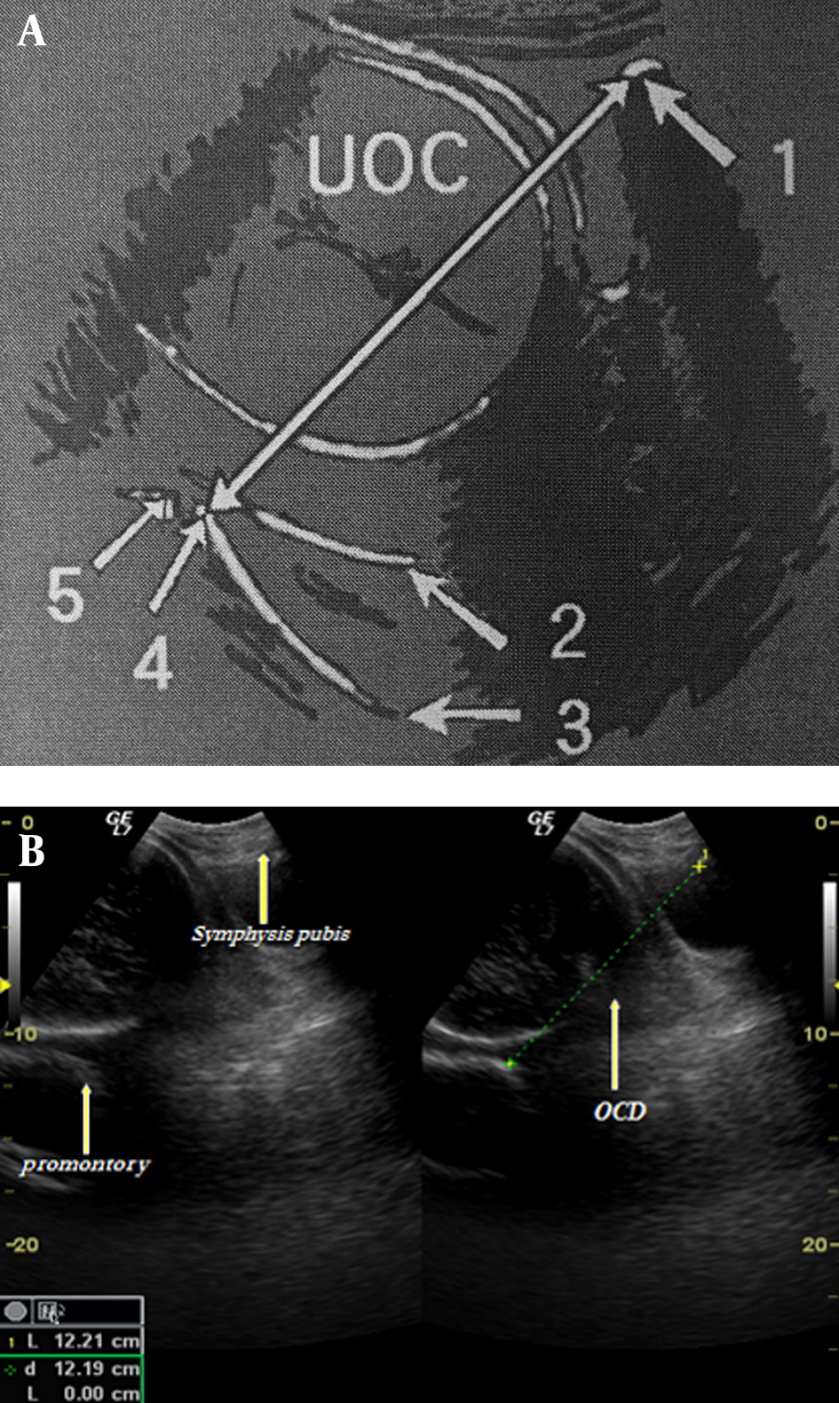
A, Schematic diagram of the pelvis and structures are used to measure the obstetric conjugate diameter (OCD) sonographically. (1: Pubic bone, 2: Posterior uterine wall, 3: Sacral periphery, 4: Promontory, 5: Fifth lumbar vertebra). B, Ultrasound picture showing bright acoustic shadow of the pubic symphysis, sacral promontory, ultrasonic obstetric conjugate, and biparietal diameter entering the pelvic inlet.

In this descriptive study, 200 pregnant women without a previous problem in the third quarter of pregnancy who were experiencing their first gestation were studied. A transabdominal US scan was done using GE Logiq Alpha 200/ Logiq 200 PRO, US machine (General Electric Medical Systems; Milwaukee, USA) with a 3.5 MHz curvilinear probe. The internal end of the superior periphery of the pubic bone to the sacral promontory was measured as the OCD (Figure 1 A and B) (6). Examination was performed in two stages (once in 25-30 weeks and then in 30-35 weeks). Factors such as maternal age, pregnancy age, fetal manifestation, accompanied diseases and type of parturition (vaginal [V] or cesarean [C]) were considered. The biparietal diameter (BPD) was measured concurrently with the measurement of OCD. Exclusion criteria included non-cephalic manifestation, pre-eclampsia, diabetes, high or low birth weight, elective cesarean, cesarean due to other reasons rather than cephalopelvic disproportion (CPD), and a BPD higher than 95% and less than 5% of the standard deviation (SD) at the same age of pregnancy (totally 56 patients were excluded; 32 in V group and 24 in C group). The SPSS software (SPSS Inc., Chicago, Illinois, USA) was used for statistical analyses. A P value < 0.05 was considered statistically significant.

## 4. Results

The mean age of the studied mothers was 24.12±4.94 years (15-40) (Table 1). On the first examination, the average OCD in groups V and C was 125.27±8.30 mm (105-144) and 112.70 ± 8.58 mm (96-135), respectively. The mean OCD in group C was significantly lower (P<0.001). At the second time, OCD in groups V and C was 125.74±8.44 mm (105-145) and 113.29±8.53 mm (97-134), respectively so that the average OCD in group C was significantly less than group V (P<0.001). The mean OCD of both sonographies in groups V and C was 125.51±8.35 (105-144.5 mm) and 112.99±8.53 (96-134.5 mm), respectively, which was significantly lower (p < 0.001) in group C. But the values of first and second OCD measurements were not significantly different (P=0.065). The optimal cut-off point of OCD in the prediction of vaginal birth was ≥ 119.75mm. The sensitivity and specificity of OCD to predict vaginal delivery was 80% and 78.5%, respectively. Based on the OCD and the number of C or V births, all cases were classified as Table 2. The OCD was significantly high in the V group (P<0.001). It is noteworthy that the causes of cesarean delivery are not determined here.

**Table 1.** Demographic Characteristics of the Study Population

Clinical Parameters	Vaginal (No=166, 83%)	Cesarean (No=34, 17%)	P Value
**Age (y)**	22.28 ± 8.44	26.36 ± 10.82	0.42
**Pregnancy age (week)**	37.24±5.12	37.36±5.24	0.52

**Table 2.** Number of Mothers Delivered V or by C Based on the Size of OCD Measured by US

Groups	Types of delivery	
	Cesarean (C)	Vaginally (V)
**Group 1: OCD ≥ 120 mm**	14 (11.5 %)	108 (88.5 %)
**Group 2: OCD < 120 mm**	51 (65.4%)	27 (34.6 %)
**Group 3: OCD < 110 mm**	24 (77.4 %)	7 (22.6 %)
**Group 4: OCD < 100 mm**	5 (100 %)	0 (0%)

## 5. Discussion

There was no significant difference in OCD between the two measurements or during pregnancy in this study (P>0.05). Moreover, Gottlicher et al. (5) demonstrated that no substantial clinically significant increase of the OCD can be found during pregnancy and in repeated pregnancy. We used the mean size of both measured as sonographic OCD value. This value was significantly less in the cesarean group compared with the vaginally delivered group (112.99±8.53 versus 125.51±8.35). Our results are consistent with the study conducted by Adadevoh et al. (7) in which the mean OCD in a group that needed cesarean was significantly less than those who were delivered vaginally. Considering the optimal cut-off point (OCD≥120 mm), the sensitivity and specificity of this method in predicting vaginal delivery was calculated as 80% and 78.5%, respectively. In this study, 118 women with an OCD greater than 120 mm and 82 with an OCD smaller than 120mm, cesarean was performed in 14 (11.9%) and 51 (62.2%) women, respectively, showing a significantly high number in the second group (P<0.001). In this regard, our results are similar to the study carried out by Katonozako et al. (6). In the current study, all the cases with an OCD smaller than 100 mm necessitated cesarean section; while in the cases with an OCD˂120 mm, vaginal birth was done in 27 cases and in the women with an OCD greater than 120 mm cesarean was required in 14 cases. As mentioned previously, the reasons for cesarean delivery were not specified clearly. Therefore, only in women with an OCD smaller than 100 mm, the necessity for cesarean could be expected strongly. We did not measure the OCD by x-ray due to ethical problems. Meanwhile, the comparative studies indicated the highly significant correlation between US and x-ray measurements for OCD (6, 8). Measuring OCD by MRI, CT scan or transvaginal sonography have been studied with acceptable results (9-11), but among all these modalities, US pelvimetry is more objective than clinical pelvimetry; more safe than x–ray and CT because of ionizing radiation absence and cheaper than MRI. In this study, despite our best effort to eliminate biases, we had no access to exact data regarding the cause(s) of cesarean surgery. So, this study cannot be considered as a study for evaluating different methods, or assessing their diagnostic value in comparison with the method studied here. Nevertheless, according to our attempt for eliminating bias, it seems that the results of this study as a preliminary study could be constructive for upcoming better and precise studies in order to evaluate the diagnostic value of OCD measured by US. Considering these points, this technique can be used as a simple, feasible, noninvasive, and inexpensive method that can be implemented at the bedside of laboring woman to assess the pelvic inlet for predicting the type of delivery.
